# Impaired Phenotype and Function of T Follicular Helper Cells in HIV-1-Infected Children Receiving ART

**DOI:** 10.1097/MD.0000000000001125

**Published:** 2015-07-13

**Authors:** Yonas Bekele, Sylvie Amu, Kidist Bobosha, Rebecka Lantto, Anna Nilsson, Birtukan Endale, Meseret Gebre, Abraham Aseffa, Bence Rethi, Rawleigh Howe, Francesca Chiodi

**Affiliations:** From Department of Microbiology, Tumor and Cell Biology, Karolinska Institutet, Stockholm, Sweden (YB, SA, RL, BR, FC); Armauer Hansen Research Institute, Addis Ababa, Ethiopia (YB, KB, BE, AA, RH); Department of Woman and Child Health, Karolinska Institutet, Stockholm, Sweden (AN); Pediatric Infectious Diseases Unit at the Pediatric Emergency Department, Astrid Lindgren's Children Hospital, Stockholm, Sweden (AN); and All Africa Leprosy, Tuberculosis and Rehabilitation Training (ALERT) Hospital, Addis Ababa, Ethiopia (MG)

## Abstract

T follicular helper (Tfh) cells are important components in development of specific humoral immune responses; whether the number and biology of Tfh cells is impaired in HIV-1-infected children is not yet studied.

The frequency, phenotype, and function of Tfh cells and B cells were determined in blood of HIV-1-infected children receiving antiretroviral therapy (ART) and age-matched controls. Flow cytometry was used to characterize the frequency of Tfh cells and B cell subsets. Cytokine expression was measured after in vitro activation of Tfh cells.

A reduced frequency of memory Tfh cells (*P* < 0.001) was identified in HIV-1-infected children and, on these cells, a reduced expression of programmed death-1 (PD-1) and inducible T cell costimulator (ICOS) (*P* < 0.001 and *P* < 0.01). Upon activation, the capacity of Tfh cells to express IL-4, an important cytokine for B cell function, was impaired in HIV-1-infected children.

B cell subpopulations in HIV-1-infected children displayed significant differences from the control group: the frequency of resting memory (RM) B cells was reduced (*P* < 0.01) whereas the frequency of exhausted memory B cells increased (*P* < 0.001). Interestingly, the decline of RM cells correlated with the reduction of memory Tfh cells (*P* = 0.02).

Our study shows that function and phenotype of Tfh cells, pivotal cells for establishment of adaptive B cell responses, are impaired during HIV-1 infection in children. A consistent reduction of memory Tfh cells is associated with declined frequencies of RM B cells, creating a novel link between dysfunctional features of these cell types, major players in establishment of humoral immunity.

## Abstract 2

Supplemental digital content is available in the text

## INTRODUCTION

T follicular helper (Tfh) cells are important components in the development of specific humoral immune responses to vaccines or microbial pathogens by participating in the formation of germinal centers (GCs) and differentiation of naïve B cells into memory B cells and plasma cells (PCs) in lymphoid tissue.^1^ Whether pathological features affecting Tfh cells may play a role in the abnormal B cell homoeostasis described to take place during HIV-1 infection in children has not been previously studied.

Tfh cells are characterized by a combination of surface markers, transcription factors, and a specific cytokine profile. They produce the cytokine IL-21 and express high levels of the chemokine receptor CXCR5, programmed death-1 (PD-1) and inducible T cell costimulator (ICOS) receptors, in addition to the transcription factor Bcl-6.^2^ Recently, additional transcription factors including IRF-4, BATE, MAF, and Ascl2 have been shown to be important for Tfh cells differentiation and function.^2,3^ Although Tfh cells are considered to be tissue resident, the presence of circulating memory Tfh cells in human peripheral blood has been shown and accepted to mirror, to some extent, tissue resident Tfh cells.^4^ Circulating Tfh cell subsets have been described in several studies using different surface marker combinations; CD27^high^CCR7^high^CD45RO^high^CXCR5^high^CCR6^high^PD-1^high^,^5^ CD45RO^+^CXCR5^+^,^6^ CXCR5^+^ with increased ICOS/PD-1,^7^ CD45RA^−^CXCR5^+^ in combination with CCR6 and CXCR3,^8^ CD45RA^−^CCR7^+^CXCR5^+^,^9^ CD45RA^−^CXCR5^+^,^10^ and CD45RO^+^CXCR5^+^CXCR3^−^PD-1^+^.^4^

The critical function of Tfh cells in formation of GCs has promoted in depth studies of these cells during HIV-1 infection. An expansion of Tfh cells in HIV-1-infected subjects that positively correlated to the frequency of GC B cells and antibody titers has been described.^11^ The presence of memory Tfh cells in circulation was shown to correlate with parameters of B cell responses induced by the stimulatory action of Tfh cells, as broad neutralizing antibodies against HIV-1 in a cohort of HIV-1-infected individuals.^4^ Another study, however, reported that Tfh cells are functionally impaired and cannot provide appropriate help to B cells.^12^ Tfh cells were shown to be increased in viremic HIV-1-infected subjects as compared with healthy subjects and to contain the highest percentage of CD4 T cells harboring HIV DNA,^13^ suggesting that Tfh cells serve as an important cellular compartment for HIV-1 infection.

Several abnormalities have been shown to affect B cells during HIV-1 infection in adults and children leading to decreased maintenance of serological memory, as measured by circulating antibodies to vaccine antigens and previously encountered pathogens.^14–16^ The frequency of resting memory (RM) B cells is reduced in blood^17–19^ of HIV-1-infected subjects and the decline of RM B cells is directly correlated to the levels of circulating antibodies against vaccination antigens (measles, pneumococcus, and tetanus).

Rare B cell populations with an exhausted phenotype have been found to be expanded in the blood of HIV-1-infected patients, including children.^19^ These populations include activated memory (AM) B cells and a low proliferating B cell type which in view of its similarity to human tonsillar B cells has been termed tissue-like memory (TLM) B cells.^20,21^ The causes for the impaired frequencies of memory B cells in the blood of HIV-1-infected patients are not fully understood; since Tfh cells provide important signals for B cell survival and differentiation it is reasonable that Tfh cells may be involved in the establishment of B cell defects during HIV-1 infection.

No previous studies have addressed whether an impairment occur in the Tfh cell compartment in HIV-1-infected children. We focused on characterizing the phenotype and function of Tfh cells in the blood of HIV-1-infected children receiving ART. Our results indicate that many HIV-1-infected children present with considerable functional and phenotypic damage of Tfh cells in spite of prolonged ART. In an attempt to increase our knowledge on how Tfh cells may contribute to the establishment of impaired B cell functions during HIV-1 infection, we related parameters of Tfh cell function to frequencies and phenotype of B cell subpopulations which in the present study displayed several abnormalities.

## MATERIALS AND METHODS

### Study Design and Subjects

A total of 103 children, 48 HIV-1-infected and 55 healthy controls, were recruited at ALERT pediatric ward and Woreda 02/03 clinic, Addis Ababa, Ethiopia and enrolled in a cross-sectional study. The recruitment took place between 2013 and 2014; HIV-1-infected patients attending the clinic, younger than 7 years of age, were consecutively included; HIV-1-infected children coinfected with either HCV or HBV (n = 5) were not included in the study. Noninfected controls were recruited to age match the HIV-1-infected children.

The clinical and immunological characteristics for the children included in the study are presented in Table 1. All HIV-1-infected children were under antiretroviral therapy (ART) and for 12 children (26.1%) treatment was initiated before 12 months of age; the children received ART for an average period of 37.0 months (range 8–66 months). Plasma HIV-1 RNA load was measured by Cobas Amplicor (Roche Molecular Systems, Branchburg, Inc., NJ). Twenty children were shown to have detectable viremia in blood (>100 copies/mL), with viral load ranging between 200 and 582,000 copies/mL; among the viremic children, 8 had a viral load higher than 10.000 copies/mL.

**TABLE 1:**
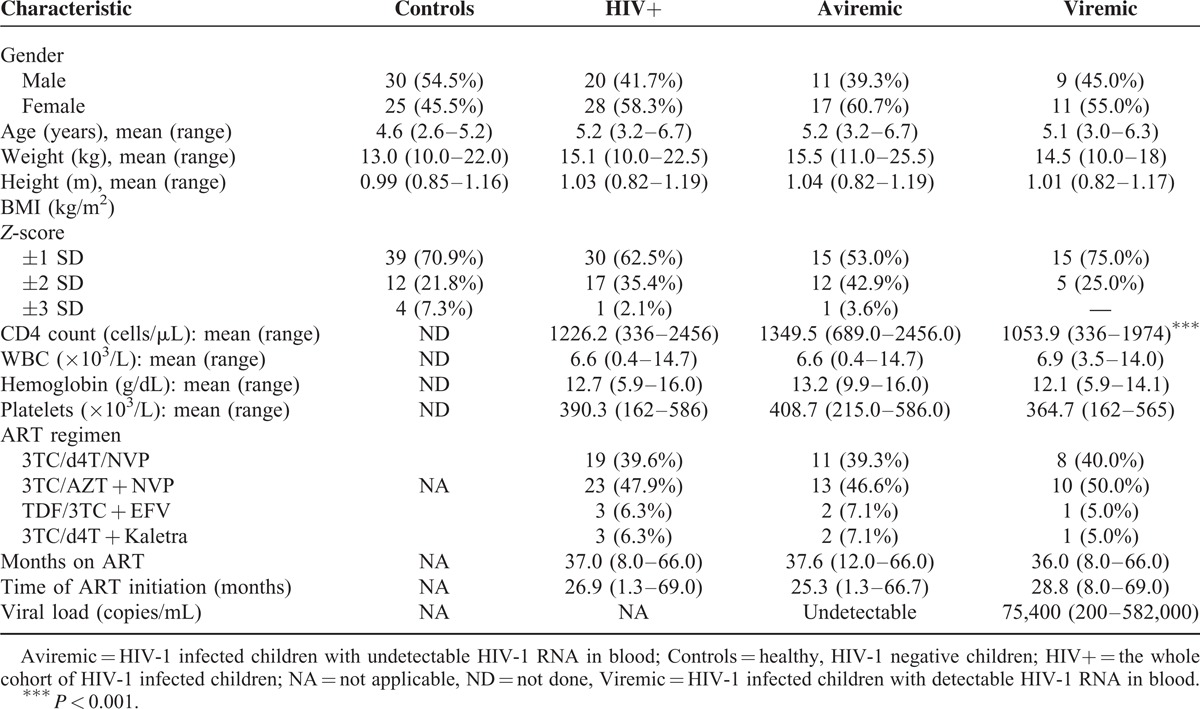
Characteristics of the Study Participants

Blood (5 mL) was collected aseptically from eligible children in EDTA containing tubes where a small aliquot (100 μL) of the whole blood was used to analyze the B cell subsets and, from the rest, peripheral blood mononuclear cells (PBMCs) were isolated and stored in RPMI containing 20% fetal bovine serum (Sigma, St. Louis MO, USA.) and 10% dimethyl sulfoxide (Sigma) in liquid nitrogen (−160°C) until further analyses were conducted.

Subpopulations of B cells were determined for 100 children. Specimens from 78 subjects, 40 healthy controls and 38 HIV-1-infected children, were used to study the distribution of Tfh cells. For 3 children we had results for the characterization of Tfh cells, but not for B cell subpopulations.

The study was approved by the AHRI/ALERT Ethics Review Committee and the National Research Ethics Committee in Ethiopia. Written informed assent was obtained from the parents or guardians of the children before commencing the study. The Ethical committee at Karolinska Institutet authorized the in vitro studies.

### Characterization of B and Tfh Cells

B cell characterization was performed using fresh whole blood. In brief, 100 μL of blood was lysed using lysing buffer (BD Biosciences, Erembodegem, Belgium). Cells were washed and the appropriate concentrations of antibodies PerCp/Cy5.5 anti-CD19 (HIB19), FITC anti-CD10 (HI10a), BV421 anti-CD21 (B-ly4), V500 anti-CD27 (M-T271), (all from BD Biosciences) in addition to Near Infra-red LIVE/DEAD Cell Stain (Molecular Probes by Life Technologies, Eugene, OR) were added. Matched isotype control antibodies were included in the analysis. The samples were fixed with 2% paraformaldehyde and acquired in the FACS Canto-II flow cytometer with FACS Diva software (BD Biosciences). Peripheral B cells were gated as CD19 + cells after exclusion of dead cells and doublets. Subpopulations of B cells were characterized according to the following markers^20^: Naive CD19+CD10–CD21+CD27–, AM CD19+CD10–CD21–CD27+, RM CD19+CD10–CD21+CD27+, and TLM CD19+CD10–CD21–CD27– B cells.

For characterization of Tfh cells, PBMCs were thawed and washed in complete medium (RPMI-1640 medium supplemented with l-glutamine, 10% FCS and penicillin–streptomycin; Thermo Scientific, South Logan, UT). Cells were then stained with the following monoclonal antibodies: V500 anti-CD4 (L200), FITC anti-CD45RO (UCHL1), PerCp/Cy5.5 anti-CXCR5 (RF8B2), BV421 anti-CD279 (PD-1; EH12.1), and PE anti-CD278 (ICOS; DX29) all from BD Biosciences and Near Infra-red LIVE/DEAD Cell Stain (Molecular Probes by Life Technologies) and analyzed.

### Intracellular Cytokine Expression in Tfh Cells

PBMCs were cultured at a concentration of 1.5 × 10^6^ cells/mL in complete medium only or stimulated with 50ng/mL PMA and 1 μg/mL Inomycin (Sigma-Aldrich, St. Louis, MO) in the presence of Golgistop (BD Biosciences) for 4 hours. The cells were first stained for cell surface markers with the monoclonal antibodies: Texas red anti-CD4 (RFT-4 g; Abcam, Cambridge, UK), FITC anti-CD45RO (UCHL1; BD Biosciences), PE-Cy7 anti-CXCR5 (J252D4; BioLegend, San Diego, CA), and Near Infra-red LIVE/DEAD Cell Stain. After washing with FACS buffer (PBS with 1%FCS) cells were fixed, permeabilized using the BD Cytofix/Cytoperm kit according to the manufacturer and stained with BV421 anti-IL-2 (5344.111), APC anti-IL-4 (MP4-25D2), BV711 anti-IFN-γ (B27) (all from BD Biosciences), and PE anti-IL-21 (3A3-N2) (eBioscience, Hatfield, UK). Cell stained with matching isotype controls were used to define appropriate positive and negative gates. Cells were fixed in 2% paraformaldehyde and the samples acquired in LSR-II flow cytometer.

### Statistical Analysis

Flow cytometry data were analyzed using Flow-Jo 9.6 (Tree Star, Ashland, OR, USA) and statistical data analysis performed with Graphpad Prism 6 (La Jolla, CA, USA). Subsets of B and T lymphocytes were compared using Mann–Whitney *U* test. Spearman rank coefficient was performed to assess the association of T and B cells’ subsets. *P* values of <0.05 were considered as statistically significant.

## RESULTS

### A Declined Frequency of Peripheral Tfh Cells in HIV-1-Infected Children

**FIGURE 1:**
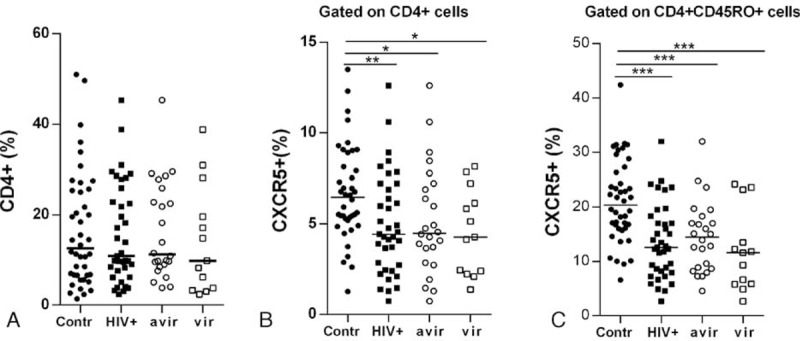
Frequencies of CD4+, Tfh and memory Tfh cells in HIV-1-infected children and control individuals. The frequencies of CD4+ T cells (A), Tfh defined as CD4+CXCR5+ T cells (B) memory Tfh cells CD4+CD45RO+CXCR5+ (C) in control individuals (n = 40), HIV-1 infected (n = 38), aviremic (n = 25), and viremic (n = 13) children are shown. ∗*P* < 0.05; ∗∗*P* < 0.01; ∗∗∗*P* < 0.001.

We defined memory Tfh cells as CXCR5+ expressing CD4+CD45RO+ T cells^8^ and the gating strategy for identification of the different Tfh subpopulations is presented in Supplementary Figure 1, http://links.lww.com/MD/A332. We measured the frequencies of CD4+ T cells, Tfh (CD4+CXCR5+) and memory Tfh cells (CD4+CD45RO+CXCR5+) in 38 HIV-1-infected children receiving ART (25 aviremic and 13 viremic) and 40 controls (Figure 1). The percentages of CD4+ T cells were comparable in both controls and infected children (with median values 12.55% vs. 10.9% and ranges 1.4–51% vs. 2.41–45.3%), independently of detectable viremia.

Interestingly, a reduced frequency of Tfh and memory Tfh cells was identified in HIV-1-infected children. The median values for the percentages of Tfh cells among CD4+ T cells were 6.46% (range 1.27–13.5%) in the controls and 4.42% (range 0.73–12.5%) in patients (*P* < 0.01), whereas the memory Tfh fractions among CD4+CD45RO+ memory T cells were 21.45% (range 6.6–42.4%) and 12.6% (range 2.7–32%) respectively (*P* < 0.001). Similar levels of Tfh and memory Tfh cells were observed in the blood of aviremic or viremic HIV-1-infected children, significantly lower than what found in controls.

The reduced frequencies of memory Tfh cells in blood of HIV-1-infected children is particularly interesting considering that frequencies of blood memory T cells are increased in this group (Supplementary Figure 2, http://links.lww.com/MD/A332).

### Tfh Cells From HIV-1-Infected Children Have a Reduced Capacity to Express IL-4

**FIGURE 2:**
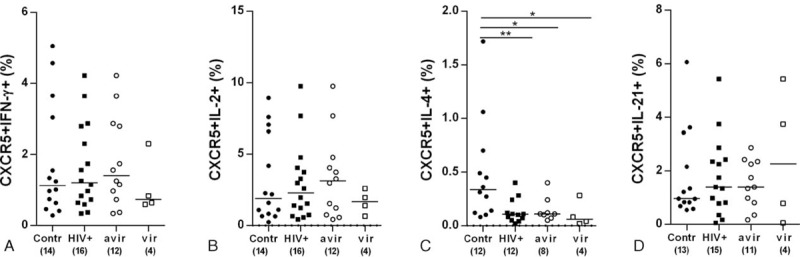
Expression of IFN-γ, IL-2, IL-4, and IL-21 in memory Tfh cells. The frequency of memory Tfh cells expressing the cytokines IFN-γ (A), IL-2 (B), IL-4 (C), and IL-21 (D) was determined after culture with PMA and Ionomycin in specimens from controls and HIV-1-infected children. The frequency of cytokine producing cells was determined after subtraction of values obtained in parallel control cultures without PMA and Ionomycin. In parenthesis the number of the analyzed specimens is shown. ∗*P* < 0.05; ∗∗*P* < 0.01.

In the next step, we studied the capacity of memory Tfh cells to express the cytokines IFN-γ, IL-2, IL-4, and IL-21 after in vitro stimulation (Figure 2). Interestingly, a smaller fraction of the memory Tfh cells from the HIV-1-infected group (both aviremic and viremic) expressed IL-4 [median value 0.3% (range 0.001–6.06%) in controls vs. 0.1% (range 0.001–5.43%) in patients; *P* = 0.01] an important cytokine for regulation of B cell functions. No significant difference was detected in the expression of IFN-γ, IL-2, and IL-21 in memory Tfh cells of the different cohorts.

The declined IL-4 expression detected in memory Tfh cells from HIV-1-infected children was confined to this cell type as CD4+ T cells, naïve and memory, did not show a different IL-4 expression after in vitro stimulation (Supplementary Figure 3, http://links.lww.com/MD/A332).

### Fewer Tfh Cells Express PD-1 and ICOS in the Blood of HIV-1-Infected Children

**FIGURE 3:**
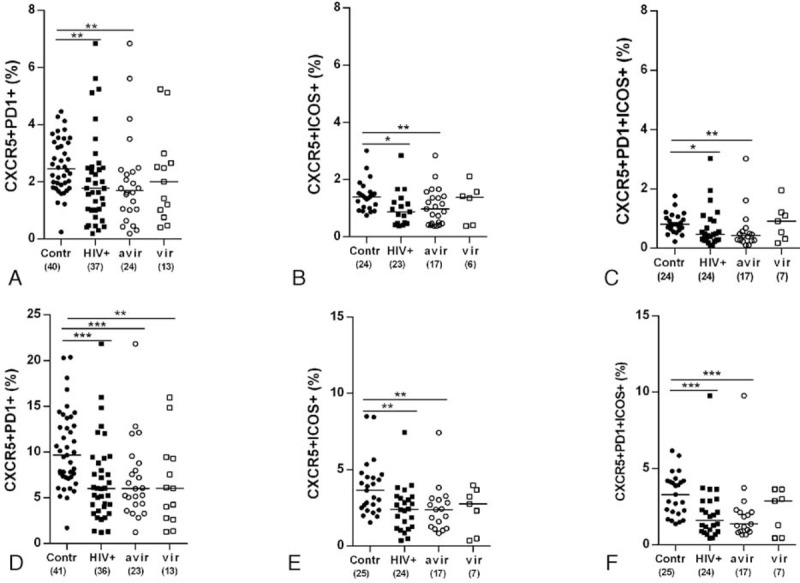
Expression of ICOS and PD-1 in CXCR5+CD4+ and memory Tfh cells. The frequency of CXCR5+ PD-1+ cells (panels A and D), CXCR5+ICOS+ cells (panels B and E) and CXCR5+ICOS+PD-1+ cells (panels C and F) were determined among gated CD4+ cells (panels A–C) or CD4+CD45RO+ cells (panels D–F). The frequencies were compared between controls and aviremic and viremic HIV-1-infected children. In parenthesis the number of the analyzed specimens is shown. ∗*P* < 0.05; ∗∗*P* < 0.01; ∗∗∗*P* < 0.001.

PD-1 and ICOS are important molecules which significantly contribute to the biology of Tfh cells. Figure 3 (Panels A–C) depicts the expression of these molecules on Tfh cells. The frequency of PD-1+, ICOS+, and PD-1+ICOS+ double positive CXCR5+ Tfh cells was analyzed among CD4+ T cells and shown to be reduced in HIV-1-infected children compared to controls (*P* = 0.01, *P* = 0.02, and *P* = 0.02, respectively).

Compared with controls, HIV-1-infected children had a lower frequency of PD-1 (*P* < 0.001), ICOS (*P* < 0.01), and PD-1+ICOS+ expressing memory Tfh (*P* < 0.001) (Figure 3D–E). The stratification of HIV-1-infected children into the 2 groups of aviremic and viremic individuals did not affect the expression of PD-1 on Tfh and memory Tfh cells (Figure 3A and D) which remained lower as compared to controls; the frequency of double positive ICOS+PD1+ Tfh cells appears to be higher, although not at a significant level, in viremic as compared to aviremic children (Figure 3B, C, E, and F).

To clarify whether the reduced frequency of cells expressing PD-1 and ICOS was only a feature of Tfh cells we examined the expression of these molecules on CD4+CD45RO+ T cells (Supplementary Figure 4, http://links.lww.com/MD/A332). Interestingly, PD-1 was similarly expressed in controls and in the whole group of HIV-1-positive children; PD-1 expression was however increased in viremic children compared to controls (*P* < 0.01) and aviremic children (*P* = 0.02). ICOS expression on CD4+CD45RO+ T cells was significantly decreased in viremic children (*P* < 0.001). The frequency of CD4+CD45RO+PD1+ICOS+ T cells was not different between controls and infected children.

### Impaired Distribution of B Cell Subpopulations in the Blood of HIV-1-Infected Children

**FIGURE 4:**
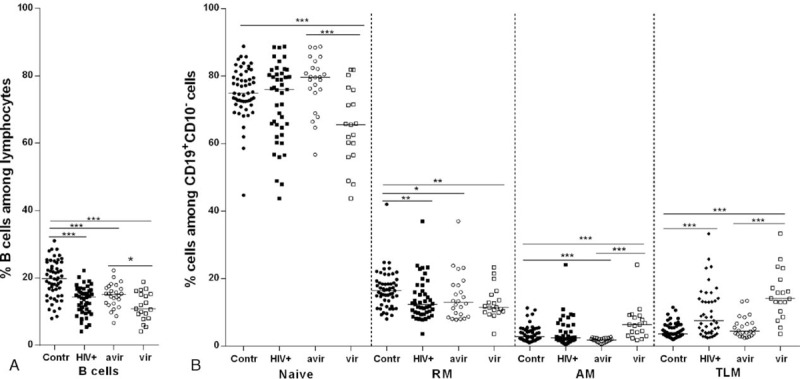
B cell subpopulations in HIV-1-infected children and controls. B cells and B cell subpopulations were characterized from whole blood of 55 controls and 45 HIV-1-infected children (26 aviremic and 19 viremic). (A) The whole peripheral B cell population was identified as CD19+ cells after exclusion of dead cells and (B) such gated cells were further delineated into 4 B cell subsets: CD19^+^CD10^−^CD21^+^CD27^−^ (naïve), CD19^+^CD10^−^CD21^−^CD27^+^ (activated memory, AM), CD19^+^CD10^−^CD21^+^CD27^+^ (resting memory, RM) and CD19^+^CD10^−^CD21^−^CD27^−^ (tissue like memory, TLM). ∗*P* < 0.05; ∗∗*P* < 0.01; ∗∗∗*P* < 0.001.

The frequencies of total B cells and B cell subpopulations (naïve, RM, AM, and TLM cells) in peripheral blood of HIV-1-infected children and controls are presented in Figure 4. We found a decreased B cell prevalence among peripheral blood lymphocytes of HIV-1-infected children as compared to noninfected children, with a median level of 14.2% (range 4.1–22.2%) for HIV-1-infected children and 19.8% (range 7.9–31%) for healthy controls (*P* < 0.001). When HIV-1-infected children were divided into viremic and aviremic, the frequency of total B cells in viremic children was shown to be further reduced as compared to aviremic children (*P* = 0.02).

The frequency of naïve B cells was not different among HIV-1-infected and control children [median 75.9% vs. 74.9% (range 43.7–88.7 vs. 44.7–88.8%) respectively] but viremic HIV-1-infected children had a reduced frequency of naïve B cells as compared to controls (*P* < 0.001) and aviremic patients (*P* < 0.001). RM B cells, an important component of serological memory, were significantly reduced in HIV-1-infected children as compared to control individuals [median 12.2% vs. 17.00% (range 3.6–37 vs. 8.0–42%); *P* < 0.01]. Both aviremic and viremic children showed a reduced frequency of RM cells compared to the controls (*P* < 0.05 and *P* < 0.01, respectively).

The median value of AM cells did not vary when comparing HIV-1-infected children and controls (2.45% vs. 2.75% respectively; ranges 0.56–24.1 vs. 1.01–11.3%). Viremic children had a significant increased frequency of AM cells compared to controls (*P* < 0.001) and aviremic patients (*P* < 0.001). HIV-1-infected children successfully responding to ART showed a frequency of AM B cells lower than the control group (*P* < 0.001). TLM B cell frequencies were increased in HIV-1-infected children [median 8.0% (range 2.4–33.3%) for HIV-1-infected and 3.6% (range 1.9–11.5%) in healthy controls; *P* < 0.001]; this increase was mostly evident in viremic children (*P* < 0.001 in relation to controls and aviremic patients).

Tfh cells have been shown to have a pivotal role for the differentiation of naïve B cells into memory B cell subpopulations in lymphoid tissue. Interestingly, the frequency of RM B cells was significantly correlated to the frequency of memory Tfh cells (r = 0.27, *P* = 0.02) in the whole group of patients and controls (Supplementary Figure 5, http://links.lww.com/MD/A332); a significant correlation was also identified among memory Tfh cells coexpressing PD-1 (r = 0.232, *P* < 0.05). This correlation lost significance when the groups of HIV-1-infected or control subjects were analyzed separately, probably due to relative small size of the cohorts.

## DISCUSSION

Increased understanding of the biology of Tfh cells may in the long run lead to improved strategies for vaccination in immunocompromised patients. In the present work, we have focused on studying the characteristics of Tfh cells which can be identified in blood during pediatric HIV-1 infection and the associations of Tfh cells with subpopulations of B cells which are profoundly impaired during HIV-1 infection in children. The purpose was to analyze the whole bulk of Tfh cells as HIV-1 infection may affect the function of Tfh cells with various specificities.

To our surprise, the frequency of memory Tfh cells in blood of HIV-1-infected children was considerably reduced; this decline did not parallel the increased frequency of memory CD4+ T cells which was detectable independently of successful treatment. We have also observed an altered cytokine profile within the memory Tfh population, which was reflected by a reduced capacity to express IL-4, a cytokine pivotal for B cell function. In a study conducted on SIV infected macaques it was shown that IL-4 production was augmented in a highly differentiated subpopulation of Tfh cells.^22^ This latter result is in contradiction with our findings of reduced IL-4 expression in memory Tfh cells from HIV-1-infected children. Defective humoral immunity in neonates was associated with limited IL-4 mediated Tfh cell responses^23^; accordingly, regulation of IL-4 expression in Tfh cells may be age, or species, dependent.

We found that the frequency of Tfh cells expressing the PD-1 and ICOS declined in HIV-1-infected children. The pivotal role that ICOS costimulatory receptor^24^ has in GCs and T-dependent antibody responses was clearly demonstrated by the severe loss of memory B cells and antibody responses experienced by ICOS-deficient individuals^25^; reduced numbers of Tfh cells were observed in the absence of ICOS^6^ and, on the other hand, overexpression of ICOS was associated with severe autoimmunity mediated by autoantibodies.^26^ ICOS may be required early in the differentiation process of a Tfh cell by induction of Bcl-6 in CD4+ T cells during dendritic cell (DC) priming, through ICOS-L provided by DCs.^27^ It was recently shown that ICOS exerts a translational control on the targeted delivery of IL-4 to cognate B cells during T-B collaborations in the GC.^28^ This latter study is interesting in relation to our results which showed a reduced expression of ICOS and IL-4 on memory Tfh cells of HIV-1-infected children, a result which may suggest that Tfh cells provide poor help for B cell differentiation during HIV-1 infection in children.

The role of PD-1/PD-1 ligands interaction in the GC in driving PC formation is being progressively elucidated. High levels of PD-1 expression on CD4(+) T cells in lymph nodes of rhesus macaques were shown to represent a valuable marker to identify Tfh cells.^29^ PD-1, generally considered as an exhaustion marker, is increasingly recognized as an important molecule to provide help for B cell differentiation in GCs. PD-1–PD-L2 interactions have recently been shown to affect the survival and maintenance of PCs in mice through a mechanism regulated by PD-1 expression on Tfh cells and PD-L2 expression on B cells.^30^ The molecular pathway leading to expression of PD-Ls on activated B cells remain poorly characterized and it is unknown whether this pathway is affected during HIV-1 infection. According to the emerging role of PD-1 as a costimulatory molecule of Tfh cells, the reduced PD-1 expression on Tfh cells of HIV-1-infected children may reflect an impaired molecular communication between Tfh cells and B cells in the GC ultimately leading to defective B cell maturation. It is interesting that, as previously reported,^31^ the frequency of PD-1+ memory CD4+CD45RO+T cells was increased during HIV-1 infection in viremic children, suggesting a specific damage to take place in PD-1 expressing memory Tfh cells which are reduced in frequency.

As previously reported by several groups,^15^ the frequency of subpopulations of memory B cells is dramatically impaired in HIV-1-infected adults and children. In the cohorts studied in the present work, a declined frequency of RM B cells was revealed in HIV-1-infected children as compared to controls. Interestingly, a direct correlation was observed between the frequencies of memory Tfh cells and RM B cells in blood of HIV-1-infected children and controls included in the study. As it is accepted that the frequency of memory Tfh cells in blood reflects the density of these cells within the lymphoid tissues and GCs,^4^ the reduced frequency of RM B cells detected in blood may, in part, be the result of a declined frequency of memory Tfh cells in the lymphoid tissue of HIV-1-infected children. On the other hand, and in view of the emerging role that GC B cells appear to have in providing differentiation signals (ICOS-L and PD-L2) to Tfh cells it cannot be firmly ruled out that damage induced by HIV-1 in the homeostasis of B cells may become a driving force for declined levels of Tfh cells during HIV-1 infection.

It is important to dissect why impaired frequencies of memory Tfh and B cell subpopulations are present in HIV-1-infected children in spite of the fact that the children were receiving ART. Detectable HIV-1 viremia was found in the blood of 20 of the 48 children included in the study (42%) and the virus titers in blood were very high in 8 (16%) of this treated children. The cause for this persistent viremia in the blood of HIV-1-infected children remains unclear but probably reflects poor compliance to treatment. The majority of HIV-1-infected children included in our study received ART after 1 year of age, and were treated for an average period of 36.9 months (range 8–66 months). It is likely that in subjects for whom ART is initiated after 1 year of age the virus may already have had adequate time to dramatically impair the function of cells, including Tfh cells, crucial for immunological responses to vaccines. In this context it is important that Tfh cells were shown to be the direct target for HIV-1 infection and to sustain elevated levels of virus replication.^13^ Initiating ART between 6 and 12 weeks of age has been shown to reduce mortality and disease progression by >70% compared to a deferred strategy.^32,33^

In conclusion, our study showed that the phenotype and function of blood memory Tfh cells are profoundly affected during HIV-1 infection in children. The strategy of providing early ART to HIV-1-infected children in less favorable economic environments should be evaluated in the context of the biology and function of Tfh cells which are pivotal for the establishment of sustained response to childhood vaccines and pathogens.
